# Mortality trends in chronic liver disease and cirrhosis from 1981 to 2015 in Taiwan

**DOI:** 10.1186/s12963-021-00269-w

**Published:** 2021-10-02

**Authors:** Shih-Yung Su, Long-Teng Lee, Wen-Chung Lee

**Affiliations:** 1 Institute of Epidemiology and Preventive Medicine, College of Public Health, National Taiwan University, Xuzhou Rd., Rm. 536, No. 17, Taipei, 100 Taiwan; 2 Innovation and Policy Center for Population Health and Sustainable Environment, College of Public Health, National Taiwan University, Taipei, Taiwan; 3 Department of Family Medicine, National Taiwan University Hospital and College of Medicine, National Taiwan University, Taipei, Taiwan; 4 Taipei Jen-Chi Hospital, Taipei, Taiwan

**Keywords:** Mortality, Chronic liver disease, Liver cirrhosis, Age–period–cohort model

## Abstract

**Background:**

Globally, the morbidity and mortality rates for chronic liver disease and cirrhosis are increasing. The National Viral Hepatitis Therapy Program in Taiwan was implemented in 2003, but evidence regarding the program’s effect on the trends of mortality for chronic liver disease and cirrhosis is limited.

**Methods:**

We analyzed mortality rates for chronic liver disease and cirrhosis in Taiwan for the period from 1981 to 2015. An autoregressive age–period–cohort model was used to estimate age, period, and cohort effects.

**Results:**

Age-adjusted mortality rates for chronic liver disease and cirrhosis all displayed a flat but variable trend from 1981 to 2004 and a decreasing trend thereafter for both sexes. The age–period–cohort model revealed differential age gradients between the two sexes; mortality rates in the oldest age group (90–94 years) were 12 and 66 times higher than those in the youngest age group (30–34 years) for men and women, respectively. The period effects indicated that mortality rates declined after 2004 in both sexes. Mortality rates decreased in men but increased in women in the 1891–1940 birth cohorts and increased in both sexes in the birth cohorts from 1950 onward.

**Conclusions:**

The National Viral Hepatitis Therapy Program in Taiwan may have contributed to the decrease in mortality rates for chronic liver disease and cirrhosis in adulthood.

**Supplementary Information:**

The online version contains supplementary material available at 10.1186/s12963-021-00269-w.

## Background

Chronic liver disease and cirrhosis can progress to hepatocellular carcinoma. Global estimates indicate that chronic liver disease and liver cirrhosis account for more than 2 and 1 million deaths annually, respectively [1]. Furthermore, the morbidity and mortality rates for chronic liver disease and cirrhosis are increasing, despite most cases being preventable [2, 3]. Together, chronic liver disease and cirrhosis were the 12th leading cause of death in the United States in 2015, accounting for 12.5 deaths per 100,000 people [4]. In Taiwan, chronic liver disease and cirrhosis constituted the tenth leading cause of death in both the sexes in 2015 (20 deaths per 100,000 people) [5].

Risk factors for chronic liver disease, cirrhosis, and liver cancer include hepatitis B virus (HBV) infection, hepatitis C virus (HCV) infection, excessive alcohol drinking, cigarette smoking, and obesity [6, 7]. In Taiwan, more than 90% of cases of end-stage liver diseases were related to HBV and HCV infection [8]. To address the growing burden of liver disease, the National HBV Vaccination Program for all infants in Taiwan was implemented in 1984 [9]. Several studies have demonstrated that the National HBV Vaccination Program has significantly reduced the incidence of and mortality from end-stage liver disease in children and adolescents born after 1984 [10–13]. Another nationwide program, namely the National Viral Hepatitis Therapy Program, was implemented in Taiwan in 2003 [9]. A study demonstrated that since its implementation, this program has reduced mortality rates for chronic liver disease, cirrhosis, and liver cancer [14]. However, the study did not consider birth cohort effects.

In the present study, we used an autoregressive age–period–cohort model to analyze mortality trends for chronic liver disease and cirrhosis in Taiwan from 1981 to 2015. Age–period–cohort analysis can help demonstrate the influence of health policy, medical technology, public hazard events, and large-scale environmental events on disease incidence and mortality rates. Accordingly, the effects of three temporal factors, namely age, period, and cohort, on mortality rates for chronic liver disease and cirrhosis as well as the effects of the National HBV Vaccination Program and Viral Hepatitis Therapy Program were discussed.

## Methods

### Data sources

All causes of death in Taiwan for the period from 1981 to 2015 were extracted from an online database provided by the Ministry of Health and Welfare. In this study, cases of mortality for chronic liver disease and cirrhosis were selected using International Classification of Diseases, Ninth Revision (ICD-9) code 571 (for the period 1981–2007), and ICD-10 codes K70, K73, and K74 (for the period after 2008). Chronic liver disease and cirrhosis were aggregated in this study because these two causes of death share the same ICD-9 code. The use of the two coding systems resulted in an estimated difference of < 3% in the numbers of cases of chronic liver disease and cirrhosis [15]. Data for 146,881 cases of mortality for chronic liver disease and cirrhosis (107,616 in men and 39,265 in women aged 30–94 years) for the period from 1981 to 2015 were collected. Patients aged younger than 30 years were not included because of the paucity of mortality cases for this age group. The data were categorized into 13 age groups (from 30–34 to 90–94 years) and 7 period groups (from 1981–1985 to 2011–2015). Midyear population data in Taiwan from 1981 to 2015 were extracted from the online database provided by the Department of Statistics of the Ministry of the Interior, in which data were similarly categorized into 13 age and 7 period groups.

### Statistical analysis

Age-specific mortality rates according to age, period, and cohort were calculated for both sexes and plotted on a log scale. Age-adjusted mortality rates for chronic liver disease and cirrhosis were calculated using the World Health Organization 2000 World Standard Population for both sexes. The average annual percentage change in mortality for chronic liver disease and cirrhosis between 1981 and 2003 (before the implementation of the National Viral Hepatitis Therapy Program) and that between 2004 and 2015 (after program implementation) were calculated. The hierarchy of age–period–cohort modeling strategy proposed by Clayton and Schifflers [16, 17] was used to evaluate the effects of age, period, and cohort through a log-linear Poisson model. All three temporal factors were included in the model. In addition, three two-factor models (namely age and period, age and cohort, and period and cohort) were considered. A likelihood ratio test was performed between the age–period–cohort model and the two-factor models to determine an appropriate model to fit the mortality data of chronic liver disease and cirrhosis. However, the age–period–cohort model exhibited nonidentifiability problem owing to the exact linear relation of the three temporal variables (cohort + age = period). A nonidentifiable age–period–cohort model typically produces an infinite set of parameter estimates with equal goodness-of-fit indices. To avoid this problem, an autoregressive age–period–cohort model was used in this study to estimate the age, period, and cohort effects [18]. Autoregressive age–period–cohort models assume a first-order autoregressive process for the cohort effects; hence, they do not have nonidentifiability problem. Autoregressive age–period–cohort models have been demonstrated to be effective in estimating disease incidence and mortality [19].

All statistical analyses were performed using SAS statistical software version 9.4 (SAS® Institute Inc, Cary, NC, USA).

## Results

**Fig. 1:**
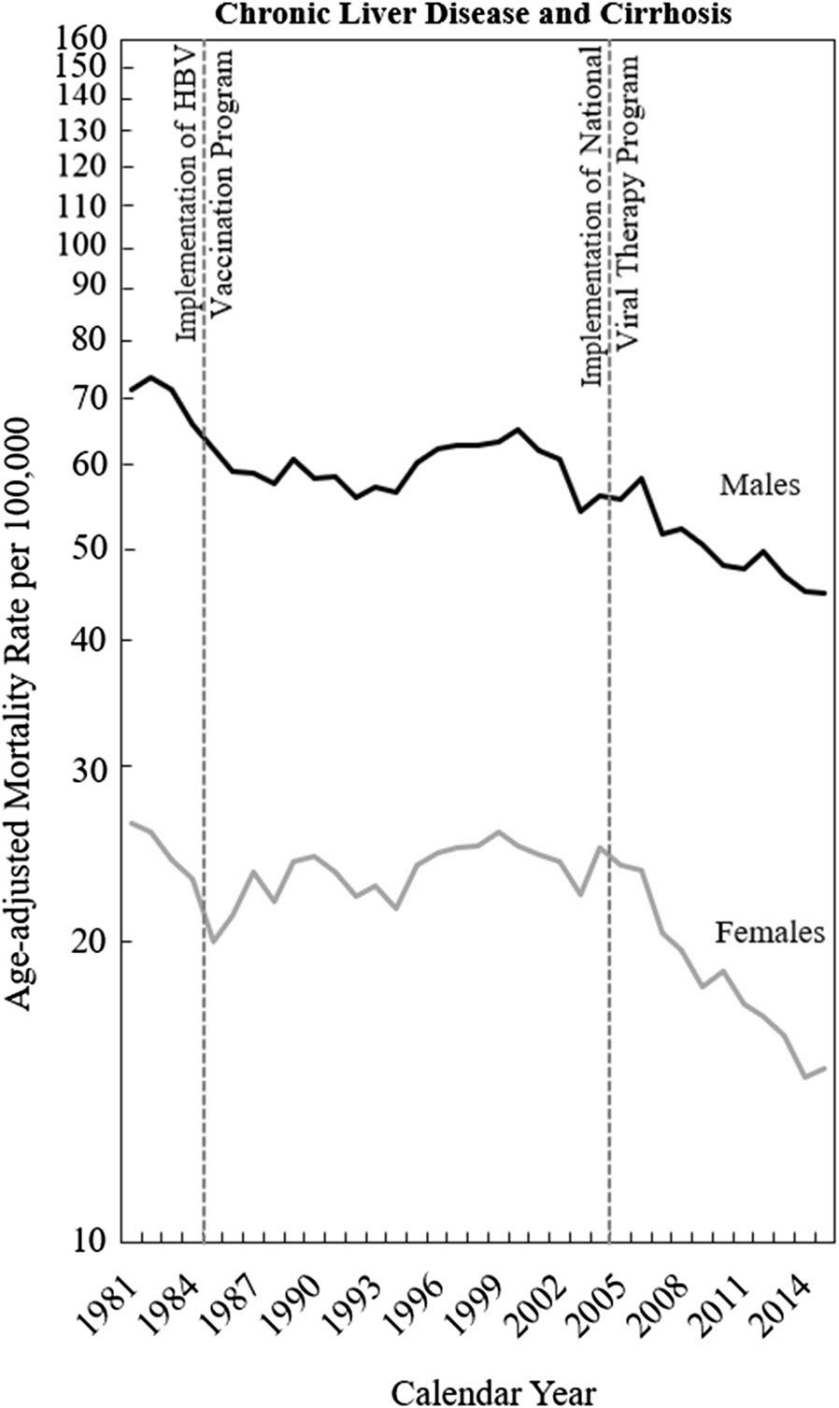
Age-adjusted mortality rates from chronic liver disease and liver cirrhosis in Taiwan

Figure 1 illustrates the secular trends in the annual age-adjusted mortality rates for chronic liver disease and cirrhosis in men and women in Taiwan from 1981 to 2015. The trends observed for both sexes were similar, but the age-adjusted mortality rate in men was considerably higher than that in women (approximately threefold). For the two sexes, we observed a flat but fluctuating trend from 1981 to 2004 and a decreasing trend thereafter; the decreasing trend was particularly pronounced in women.

Table 1 presents the average annual percentage change in mortality for chronic liver disease and cirrhosis between 1981 and 2003 and that between 2004 and 2015 in Taiwan. For men, a slight yearly decrease in age-adjusted mortality was observed from 1981 to 2003 (− 0.56% per annum), and a more pronounced decrease was recorded from 1981 to 2003 (− 2.28% per annum). For women, a slight increase in age-adjusted mortality was observed between 1981 and 2003 (0.12% per annum), followed by a decrease from 2004 to 2015 (− 4.82% per annum). The trends in mortality rates were consistent across all age groups for men (except for the 75–89-year age groups) and women (except for the 45–49-year age group).

**Table 1:** The average annual percentage change of age-adjusted rates and age-specific rates of chronic liver disease and cirrhosis between 1981 and 2003, and between 2004 and 2015 for men and women in Taiwan

	Men			Women		
	Between 1981 and 2003 (%)	Between 2004 and 2015 (%)	Change* (%)	Between 1981 and 2003 (%)	Between 2004 and 2015 (%)	Change* (%)
Age-adjusted rates	− 0.56	− 2.28	− 1.73	0.12	− 4.82	− 4.94
*Age-specific rates*						
30–34	2.30	− 4.39	− 6.69	1.60	− 4.15	− 5.75
35–39	2.20	− 2.62	− 4.83	0.04	− 1.35	− 1.39
40–44	1.07	− 1.57	− 2.63	− 0.92	− 4.55	− 3.64
45–49	0.35	− 0.50	− 0.85	− 0.91	− 0.25	0.66
50–54	0.20	− 0.97	− 1.17	− 1.42	− 3.99	− 2.57
55–59	0.10	− 1.35	− 1.45	0.21	− 5.38	− 5.59
60–64	− 0.26	− 6.09	− 5.83	− 0.53	− 9.43	− 8.91
65–69	− 1.90	− 4.70	− 2.79	0.12	− 6.64	− 6.76
70–74	− 2.37	− 3.98	− 1.61	0.72	− 4.55	− 5.28
75–79	− 3.18	− 2.81	0.37	1.23	− 4.28	− 5.50
80–84	− 3.56	− 2.23	1.32	1.31	− 2.67	− 3.98
85–89	− 3.10	− 2.64	0.46	1.91	− 3.22	− 5.13
90–94	− 3.23	− 5.67	− 2.45	1.94	− 3.29	− 5.23

*Change = (average annual percentage change between 2004 and 2015) − (average annual percentage change between 1981 and 2003)

**Fig. 2:**
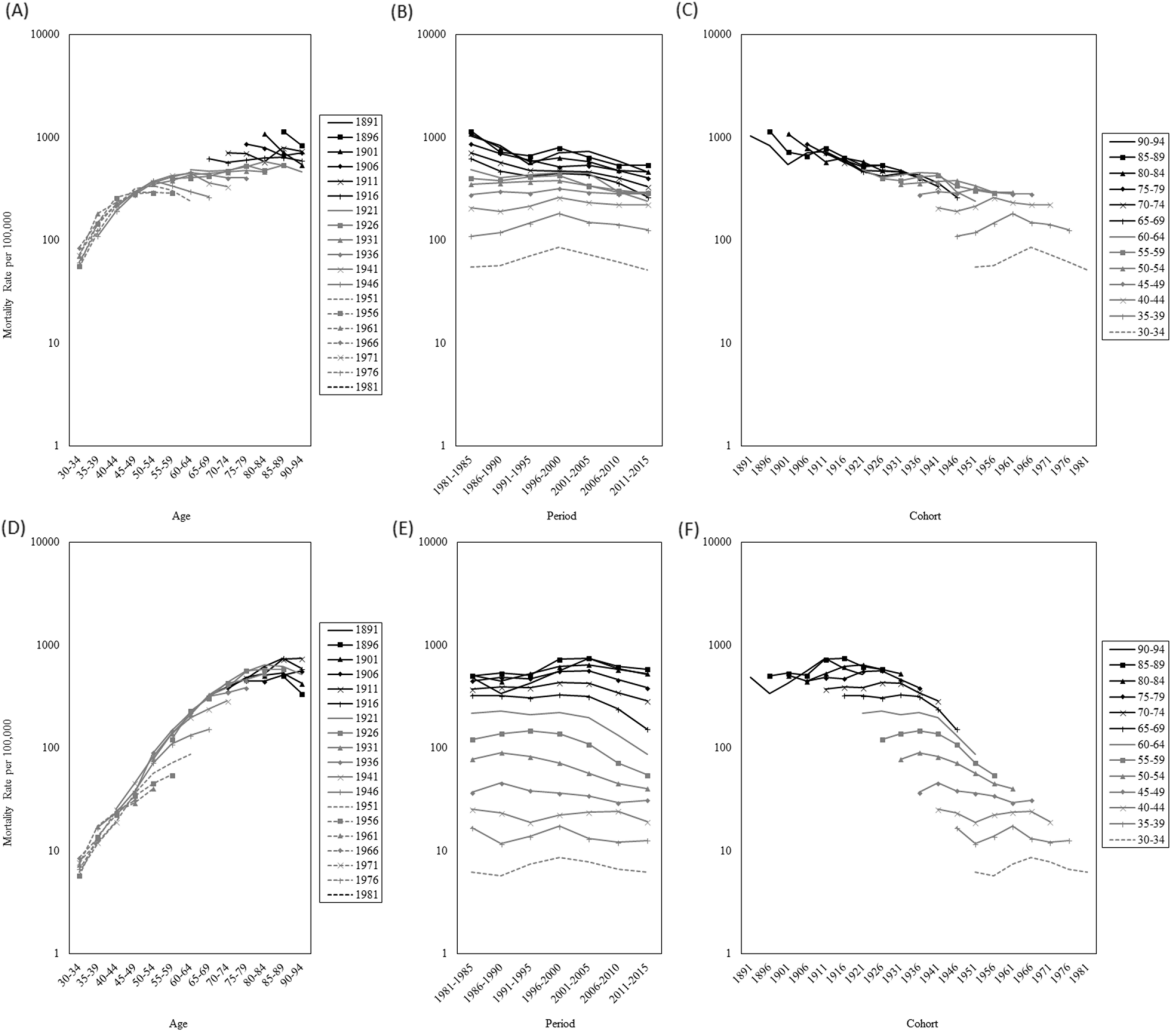
Age-specific mortality rates from chronic liver disease and liver cirrhosis in Taiwan. Upper panels (**A**–**C**) for men and lower panels (**D**–**F**) for women

Figure 2 presents a simple graphical depiction of chronic liver disease and cirrhosis data for men (upper panels) and women (lower panels). The data for men indicated that mortality rates for chronic liver disease and cirrhosis in the oldest age group (90–94 years) were approximately 20 times higher than those in the youngest age group (30–34 years) (Fig. 2A). The period trend for men (Fig. 2B) revealed that mortality rates increased from 1981 to 2000 and decreased after 2000 in the younger age groups (30–59 years). In the older age groups (60–94 years), mortality rates exhibited a long-term decreasing trend from 1981 to 2015. The birth cohort data for men (Fig. 2C) showed that mortality trends decreased slowly from 1891 to 1946, increased drastically from 1946 to 1966, and decreased thereafter. The data for women indicated that mortality rates for chronic liver disease and cirrhosis in the oldest age group (90–94 years) were approximately 80 times higher than those in the youngest age group (30–34 years) (Fig. 2D). The period trend for women (Fig. 2E) revealed that mortality rates were flat but fluctuating in the younger age groups (30–49 years). In the older age groups (50–94 years), mortality rates exhibited a slightly increasing trend initially, followed by a decreasing trend. The birth-cohort trends for women (Fig. 2F) demonstrated that mortality rates increased slowly from 1891 to 1941, decreased drastically from 1941 to 1951, and leveled off after 1951.

Table 2 presents the likelihood ratio test results for the model hierarchy. For men, the age–period–cohort model exhibited the lowest deviance (436.05). The likelihood ratio test results for the one-factor models and two-factor models all exhibited significantly higher deviance than did the results for the age–period–cohort model. For women, the age–period–cohort model exhibited the lowest deviance (129.29), and all other models exhibited significantly higher deviance. The likelihood ratio test results indicate that all three temporal variables should be considered for both sexes when modeling mortality rates for chronic liver disease and cirrhosis.

**Table 2:** Age–period–cohort model for chronic liver disease and liver cirrhosis mortality rates in Taiwan

Models	df	Deviance	Likelihood ratio statistic (df*)	*P* value
*Males*				
Age	78	3013.15	2577.10 (23)	< 0.0001
Period	84	33,327.97	32,891.92 (29)	< 0.0001
Cohort	72	11,177.27	10,741.22 (17)	< 0.0001
AP	72	1470.36	1034.31 (17)	< 0.0001
AC	60	1155.41	719.36 (5)	< 0.0001
PC	66	6502.79	6066.74 (11)	< 0.0001
APC	55	436.05	Reference	Reference
*Females*				
Age	78	2045.93	1916.64 (23)	< 0.0001
Period	84	60,272.85	60,143.56 (29)	< 0.0001
Cohort	72	11,457.84	11,328.55 (17)	< 0.0001
AP	72	812.07	682.78 (17)	< 0.0001
AC	60	450.82	321.53 (5)	< 0.0001
PC	66	1265.24	1135.95 (11)	< 0.0001
APC	55	129.29	Reference	Reference

df: degree of freedom; Likelihood ratio statistic: increase in deviance from the APC model; df*: increase in df from the APC model; APC: full age–period–cohort model; AC: age–cohort model; AP: age–period model; PC: period–cohort model

**Fig. 3:**
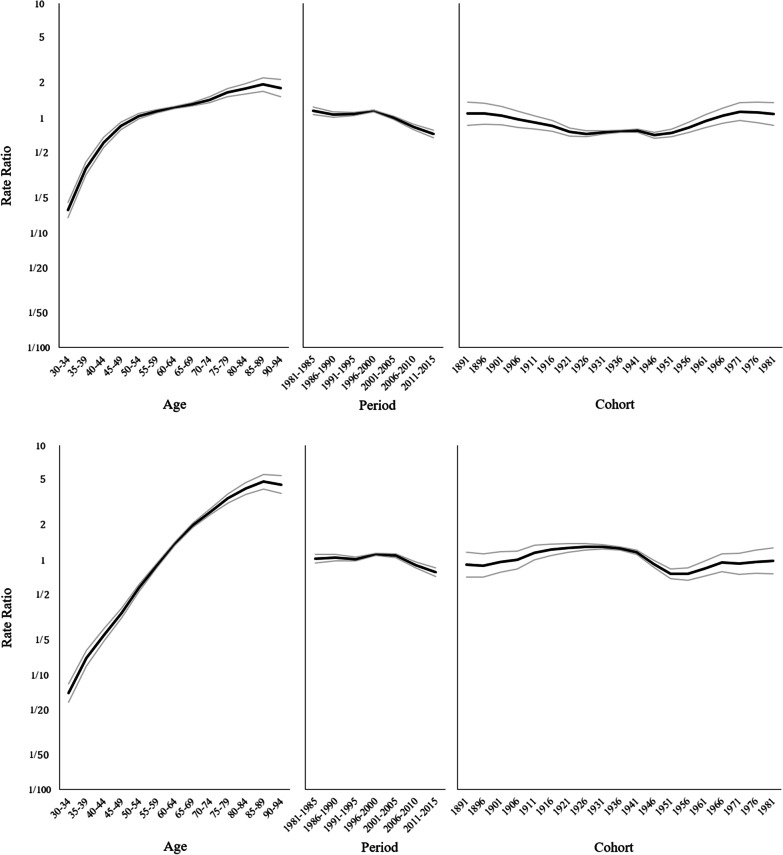
Age, period, and cohort effects of chronic liver disease and liver cirrhosis mortality in Taiwan. Upper panels for men and lower panels women

Figure 3 displays the results of age–period–cohort modeling for men (upper panels) and women (lower panels). For men, mortality rates were 12 times higher in the oldest age group (90–94 years) than in the youngest age group (30–34 years). The period effect exhibited a flat but slightly variable trend from 1981 to 2000 and a decreasing trend thereafter. Moreover, the relative risk of mortality for chronic liver disease and cirrhosis from 2011 to 2015 was 37% lower than that from 1996 to 2000. The cohort effect decreased slightly from 1891 to 1946, increased from 1946 to 1971, and leveled off in the recent birth cohorts. For women, mortality rates were 66 times higher in the oldest age group (90–94 years) than in the youngest age group (30–34 years). The period effect exhibited a slightly increasing trend from 1981 to 2000, followed by a decreasing trend. Furthermore, the relative risk of mortality for chronic liver disease and cirrhosis from 2011 to 2015 was 30% lower than that from 1996 to 2000. The cohort effect exhibited an increasing trend from 1891 to 1941, a suddenly decreasing trend from 1941 to 1951, and an increasing trend after 1951.

## Discussion

This study revealed that mortality rates for chronic liver disease and cirrhosis in the oldest age group (90–94 years) were 12 and 66 times higher than those in the youngest age group (30–34 years) for men and women, respectively. In a study on mortality rates for hepatocellular carcinoma in Taiwan, similar differential age gradients were observed in men and women [19]. Chronic liver disease and cirrhosis can progress to hepatocellular carcinoma, with a 5-year progression risk of approximately 22–28% [20]. Furthermore, common risk factors for chronic liver disease, cirrhosis, and hepatocellular carcinoma include hepatitis virus infection, alcohol consumption, and metabolic conditions. These common risk factors may partially explain the similarity in mortality rate age gradients for chronic liver disease, cirrhosis, and hepatocellular carcinoma. However, for both sexes, the age gradients in chronic liver disease and cirrhosis mortality rates in Taiwan are lower than the age gradients in Canada and Europe [21, 22]; this warrants further investigation.

When estimating the birth cohort effect, we noted that mortality rates for chronic liver disease and cirrhosis decreased in men but increased in women in the 1891–1940 birth cohorts. The rates increased in both sexes in the birth cohorts from 1950 onward. Men and women in the 1891–1940 birth cohorts experienced the era of Japanese occupation (1895–1945) and the early postwar period (1945–1949) during their childhood. Living in a changing society may have caused multiple adverse health outcomes. Men and women in the birth cohorts after 1950 experienced industrialization, westernization, urbanization, globalization, and economic growth during their childhood. Previous studies have demonstrated that rapid economic growth and income growth are associated with obesity and high alcohol consumption [23–26]. This may explain in part the increase in male and female mortality rates in these birth cohorts.

This study showed that mortality rates for chronic liver disease and cirrhosis declined after 2004 in both sexes (see Fig. 1, Table 1, and the period effects in Fig. 3). The decrease may have stemmed from the National Viral Hepatitis Therapy Program in Taiwan. This program was launched in 2003 by the National Health Insurance Administration, which provides nearly 100% health coverage for the Taiwanese population, to reach all patients infected with HBV and HCV [9]. This program reimburses treatments, including lamivudine, interferon α, pegylated interferon α, entecavir, telbivudine, tenofovir, adefovir, and direct-acting antiviral agents, for patients with viral hepatitis infections. In the early 1980s, approximately 15% to 20% of Taiwanese adults tested positive for HBV surface antigen [27, 28], but only approximately 1% to 2.5% exhibited HCV infection [29–31]. However, antiviral therapy for HBV infection was reported to be less effective than that for HCV infection [32]. The effect of antiviral therapy in eliminating HBV has been reported to be limited [33, 34]. Another study indicated that the antiviral therapy program was ineffective in reducing mortality for acute and chronic viral hepatitis in Taiwan [35]. Although the role of antiviral therapy in eliminating HBV was unclear, several studies have demonstrated that the antiviral therapies lamivudine, entecavir, and telbivudine could substantially reduce inflammation and fibrosis in the liver, prevent liver failure for patients with liver cirrhosis, and significantly reduce mortality for liver cirrhosis and liver cancer [34, 36–38]. Our study results are consistent with those of a previous study in which an age–period model indicated that the program may have reduced mortality rates for chronic liver disease and cirrhosis in Taiwan [14].

The possibility of chronic liver disease and cirrhosis progressing to hepatocellular carcinoma is high. A previous study using the same autoregressive age–period–cohort model as the present study reported an increasing trend for hepatocellular carcinoma mortality from 1976 to 2005 [19]. However, the data collection period ended in 2005, and conclusions could not be drawn on whether the trend would continue to increase or start declining after 2005. We performed additional analyses to examine the effect of the National Viral Hepatitis Therapy Program on liver cancer mortality. The age-adjusted liver cancer mortality rates (30 to 94 years old) displayed increasing trends from 1981 to 2003 and decreasing trends from 2004 to 2015 for both sexes (Additional file 1: Figure S1). Similar findings were also noted in age-specific mortality rates for both sexes (Additional file 1: Table S1). These findings are also consistent with those of a previous study [14]. We also performed additional analyses to examine the effect of the National Viral Hepatitis Therapy Program on the age distribution of chronic liver disease and cirrhosis mortality. The same autoregressive age–period–cohort model was used to analyze the data before and after the implementation of the nationwide program. The results indicated that for both sexes, the age effects before program implementation were similar to those after program implementation (Additional file 1: Figure S2).

Taiwan was previously a hyperendemic region for HBV infection [39, 40]. The National HBV Vaccination Program for all infants born after 1984 has converted Taiwan into a low-endemic region [41]. The reduction of mortality rates for chronic liver disease and cirrhosis observed in our study was not attributable to the National HBV Vaccination Program, because our abstracted data only included birth cohorts from 1891 to 1981. Those vaccinated as infants in 1984 turned 30 years old in 2015 at the earliest, and individuals aged younger than 30 years old precluded from this study due to the paucity of mortality cases at this age. A further reduction in mortality for chronic liver disease and cirrhosis can be anticipated when the vaccine-protected cohorts reach middle and late adulthood.

The successful vaccination program and the antiviral therapy program in Taiwan suggest that other nonviral risk factors, such as cigarette smoking, alcohol consumption, and obesity, may become the next principal targets to control chronic liver disease, liver cirrhosis, and hepatocellular carcinoma. The prevalence of cigarette smoking in adulthood decreased from 2009 to 2015 for both sexes according to a report from the Taiwanese Adult Smoking Behavior Surveillance System [42]. From 2009 to 2013, the prevalence of alcohol consumption decreased slightly for men but increased for women, according to a report from the National Health Interview Survey [43]. The same report also indicated that the prevalence of high triglyceride levels increased from 2001 to 2009 for both sexes [43]. Excessive alcohol drinking is a major cause of liver‐related death, accounting for approximately three-quarters of deaths in the USA and Europe [6, 44]. A recent study revealed that fatty liver, high triglyceride levels, and diabetes mellitus history were significantly associated with hepatocellular carcinoma in patients without HBV or HCV infection in Taiwan [45]. These nonviral risk factors have been demonstrated to be critical determinants for hepatocellular carcinoma in other low-endemic HBV regions, such as the UK and the USA [46, 47]. Because Taiwan is also becoming a low-endemic region for HBV infection, nonviral risk factors for chronic liver disease, liver cirrhosis, and hepatocellular carcinoma warrant attention.

## Conclusion

This study analyzed mortality rates for chronic liver disease and cirrhosis in Taiwan from 1981 to 2015. The National Viral Hepatitis Therapy Program may have contributed to the decrease in mortality rates for chronic liver disease and cirrhosis in adulthood.

## Supplementary Information

**Additional file 1.Figure S1.** The age-adjusted liver cancer mortality rates from 1981 to 2015. **Figure S2.** The age effects from autoregressive age–period–cohort analysis of chronic liver disease and cirrhosis mortality for two sexes before and after implementation of national hepatitis therapy program in Taiwan. **Table S1.** The average annual percentage change of age-adjusted rates and age-specific rates of liver cancer between 1981 and 2003, and between 2004 and 2015 for men and women in Taiwan.

## Data Availability

The datasets used and/or analyzed during the current study are available from the corresponding author on reasonable request.

## Abbreviations

HBV: Hepatitis B virus
HCV: Hepatitis C virus
