# Supplementary material for: Mortality trends in chronic liver disease and cirrhosis from 1981 to 2015 in Taiwan
Source: Popul Health Metr. 2021 Oct 2;19:36. doi: 10.1186/s12963-021-00269-w (PMC8487474; doi:10.1186/s12963-021-00269-w)
Supplement: Supplementary file 1 — Additional file 1.Figure S1. The age-adjusted liver cancer mortality rates from 1981 to 2015. Figure S2. The age effects from autoregressive age–period–cohort analysis of chronic liver disease and cirrhosis mortality for two sexes before and after implementation of national hepatitis therapy program in Taiwan. Table S1. The average annual percentage change of age-adjusted rates and age-specific rates of liver cancer between 1981 and 2003, and between 2004 and 2015 for men and women in Taiwan. [file 12963_2021_269_MOESM1_ESM.docx]

**Supplementary material**


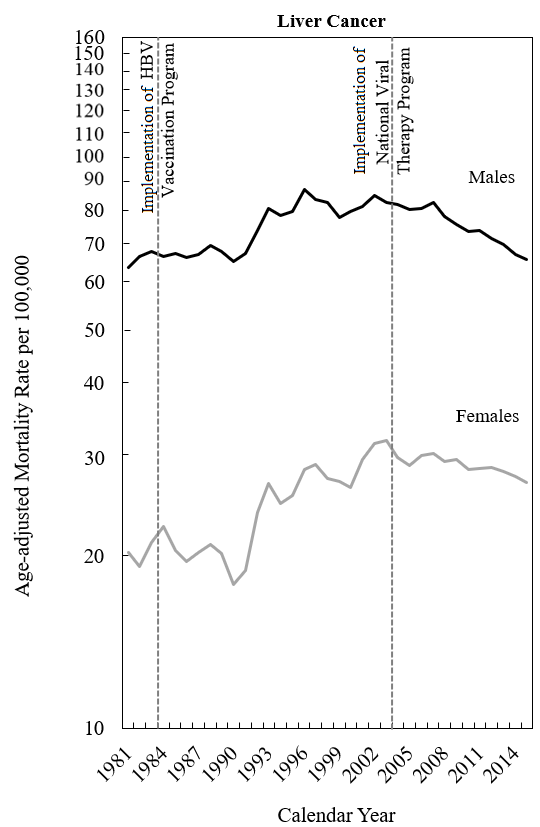


Figure S1. The age-adjusted liver cancer mortality rates from 1981 to 2015.

Table S1. The average annual percentage change of age-adjusted rates and age-specific rates of liver cancer between 1981 and 2003, and between 2004 and 2015 for men and women in Taiwan.

|  | Men | | |  | Women | | |
| --- | --- | --- | --- | --- | --- | --- | --- |
|  | Between 1981 and 2003 | Between 2004 and 2015 | Change* |  | Between 1981 and 2003 | Betweenn 2004 and 2015 | Change* |
| Age-adjusted rates | 1.36% | -2.04% | -3.40% |  | 2.33% | -0.83% | -3.15% |
| Age-specific rates |  |  |  |  |  |  |  |
| 30-34 | -0.19% | -7.75% | -7.56% |  | -1.12% | -3.42% | -2.30% |
| 35-39 | -0.70% | -7.09% | -6.39% |  | -2.95% | -4.50% | -1.55% |
| 40-44 | -0.59% | -3.98% | -3.39% |  | -3.64% | -2.34% | 1.30% |
| 45-49 | -0.77% | -2.97% | -2.21% |  | -3.12% | -4.03% | -0.91% |
| 50-54 | 0.31% | -2.92% | -3.23% |  | -1.11% | -2.87% | -1.75% |
| 55-59 | 1.48% | -3.05% | -4.53% |  | 0.51% | -2.75% | -3.26% |
| 60-64 | 2.46% | -3.87% | -6.33% |  | 2.28% | -4.01% | -6.28% |
| 65-69 | 2.42% | -2.71% | -5.13% |  | 2.94% | -2.64% | -5.59% |
| 70-74 | 2.13% | -1.26% | -3.39% |  | 4.44% | -0.39% | -4.82% |
| 75-79 | 1.80% | 2.17% | 0.38% |  | 4.20% | 0.89% | -3.30% |
| 80-84 | 3.07% | 0.99% | -2.07% |  | 4.62% | 1.85% | -2.78% |
| 85-89 | 2.29% | 0.49% | -1.79% |  | 4.96% | 3.45% | -1.51% |
| 90-94 | -0.75% | 0.17% | 0.92% |  | 5.56% | 1.19% | -4.37% |

*change = (average annual percentage change between 2004 and 2015) – (average annual percentage change between 1981 and 2003).


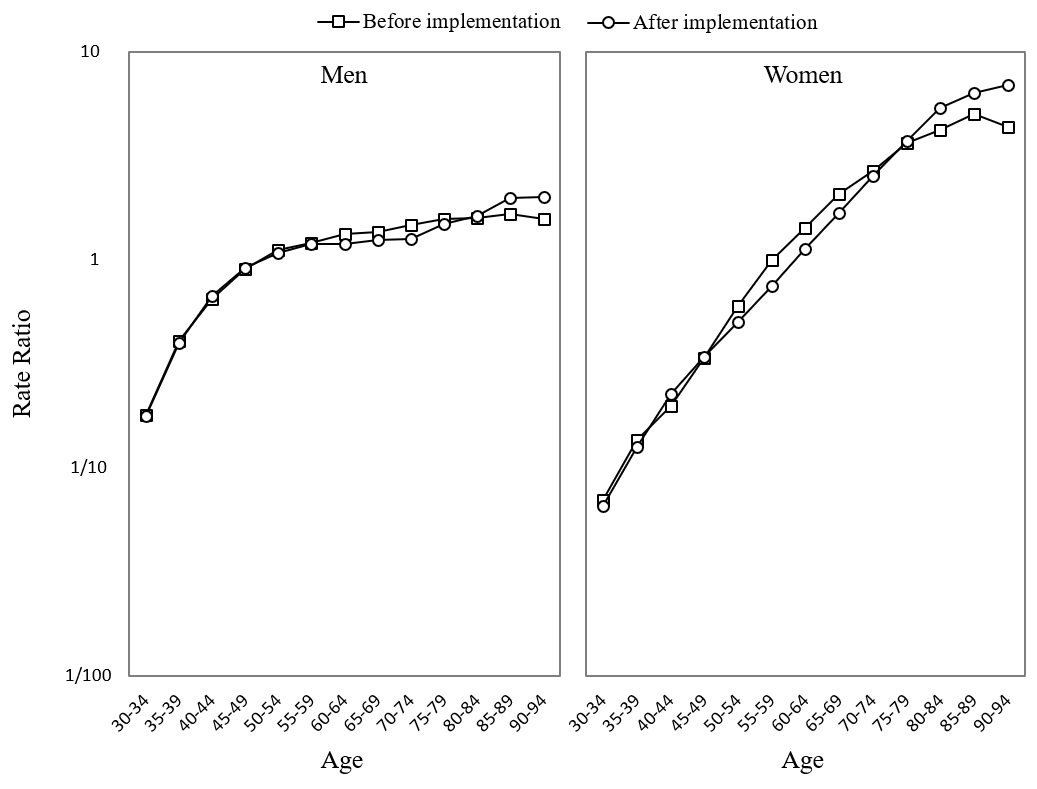


Figure S2. The age effects from autoregressive age-period-cohort analysis of chronic liver disease and cirrhosis mortality for two sexes before and after implementation of national hepatitis therapy program in Taiwan
